# Identification of Novel *FBN2* Variants in a Cohort of Congenital Contractural Arachnodactyly

**DOI:** 10.3389/fgene.2022.804202

**Published:** 2022-03-10

**Authors:** Liying Sun, Yingzhao Huang, Sen Zhao, Wenyao Zhong, Jile Shi, Yang Guo, Junhui Zhao, Ge Xiong, Yuehan Yin, Zefu Chen, Nan Zhang, Zongxuan Zhao, Qingyang Li, Dan Chen, Yuchen Niu, Xiaoxin Li, Guixing Qiu, Zhihong Wu, Terry Jianguo Zhang, Wen Tian, Nan Wu

**Affiliations:** ^1^ Department of Hand Surgery, Clinical and Research Center for Congenital Hand Deformities and Rare Diseases, Beijing Jishuitan Hospital, Beijing, China; ^2^ Department of Orthopedic Surgery, State Key Laboratory of Complex Severe and Rare Diseases, Peking Union Medical College Hospital, Peking Union Medical College and Chinese Academy of Medical Sciences, Beijing, China; ^3^ Beijing Key Laboratory for Genetic Research of Skeletal Deformity, Beijing, China; ^4^ Key Laboratory of Big Data for Spinal Deformities, Chinese Academy of Medical Sciences, Beijing, China; ^5^ State Key Laboratory of Complex Severe and Rare Diseases, Medical Research Center, Peking Union Medical College Hospital, Peking Union Medical College and Chinese Academy of Medical Sciences, Beijing, China

**Keywords:** FBN2 (fibrillin-2), congenital contractural arachnodactyly, arthrogryposis, novel variants, clinical genetics, musculo-skeletal diseases

## Abstract

Congenital contractural arachnodactyly (CCA) is a rare autosomal dominant disorder of connective tissue characterized by crumpled ears, arachnodactyly, camptodactyly, large joint contracture, and kyphoscoliosis. The nature course of CCA has not been well-described. We aim to decipher the genetic and phenotypic spectrum of CCA. The cohort was enrolled in Beijing Jishuitan Hospital and Peking Union Medical College Hospital, Beijing, China, based on Deciphering disorders Involving Scoliosis and COmorbidities (DISCO) study (http://www.discostudy.org/). Exome sequencing was performed on patients’ blood DNA. A recent published CCA scoring system was validated in our cohort. Seven novel variants and three previously reported *FBN2* variants were identified through exome sequencing. Two variants outside of the neonatal region of *FBN2* gene were found. The phenotypes were comparable between patients in our cohort and previous literature, with arachnodactyly, camptodactyly and large joints contractures found in almost all patients. All patients eligible for analysis were successfully classified into likely CCA based on the CCA scoring system. Furthermore, we found a double disease-causing heterozygous variant of *FBN2* and *ANKRD11* in a patient with blended phenotypes consisting of CCA and KBG syndrome. The identification of seven novel variants broadens the mutational and phenotypic spectrum of CCA and may provide implications for genetic counseling and clinical management.

## Introduction

Congenital contractural arachnodactyly (CCA), also known as Beals syndrome, is an autosomal dominantly inherited connective tissue disorder with an unknown prevalence (Putnam et al., 1995). The clinical manifestations of CCA primarily include arachnodactyly, congenital joint contractures, crumpled ears, kyphoscoliosis, chest deformity, dolichostenomelia, muscle hypoplasia, micrognathia and high arched palate (Guo et al., 2016; Meerschaut et al., 2019). CCA is caused by variants in fibrillin-2 (*FBN2*) gene. Fibrinllin-2 is an integral component of elastin fibers in extracellular matrix (ECM), which provides supporting structure for tissues and scaffolds for physiological processes (Olivieri et al., 2010). Fibrillin-2 mediates signaling molecules on cell surfaces, including transforming growth factor *β*(TGF-*β*), bone morphogenetic proteins (BMPs), integrins and controls ECM formation and remodeling. Pathogenic variants in *FBN2* may weaken microfibril structure or disrupt binding capability, subsequently weaken the elastic fiber and perturbate ECM-mediated signaling, which leads to the anomalies of CCA (Ratnapriya et al., 2014).

Thus far, only 91 variants in *FBN2* gene associated with CCA have been described, as listed in the Human Genome Mutation Database (HGMD). Most of these variants cluster in a hotspot region, which is known as neonatal region, spanning from exon 23 to exon 35 (Meerschaut et al., 2019), which encodes the calcium-binding epidermal growth factor-like (cbEGF) domains. However, a limited number of variants outside the neonatal region has also been reported (Callewaert et al., 2009).

Recently, a clinical scoring system for CCA was developed to classify patients into likely CCA or unlikely CCA, which was based on the presence or absence of the ten main clinical features of this disorder (Meerschaut et al., 2019). Developing a scoring system is particularly important due to the phenotype overlap between CCA and other connective tissue disorders like Marfan syndrome and type VI collagenopathies (Also named as Bethlem myopathy) (Bamshad et al., 1996). However, this scoring system has not been tested in independent cohort and the genotype-phenotype correlation of CCA is still elusive.

In this study, we identified ten pathogenic *FBN2* variants in 27 CCA patients from ten families, of which seven are novel variants. We provide their clinical manifestations and speculate variants’ impact on protein function based on variant location. Furthermore, we validated the clinical utility of a newly developed scoring system for CCA (Meerschaut et al., 2019).

## Materials and Methods

### Participants

We included ten families diagnosed with CCA and carrying pathogenic *FBN2* variant from Beijing Jishuitan Hospital and Peking Union Medical College Hospital based on the Deciphering disorders Involving Scoliosis and COmorbidities (DISCO) study (http://www.discostudy.org/) (Tian et al., 2020; Sun et al., 2021). Informed consent was obtained from all patients or their guardians. This study was approved by the institutional review board at Beijing Jishuitan Hospital and Peking Union Medical College Hospital.

### Genetic Testing

As a part of DISCO study, peripheral blood DNA from probands and available familial members were prepared into Illumina paired-end libraries and underwent whole-exome capture with the Agilent V5, followed by sequencing on the Illumina HiSeq 4,000 platform (Illumina, San Diego, CA, United States). In-house-developed Peking Union Medical college hospital Pipeline (PUMP) was used for variant calling and annotation (Zhao et al., 2020; Chen et al., 2021; Sun et al., 2021).

Variant-encoding amplicons were amplified by PCR from genomic DNA obtained from subjects and purified using an Axygen AP-GX-50 kit (lot no. 05915KE1) and conducted Sanger sequencing on an ABI3730XL instrument.

### ES Data Interpretation

Rare variants with minor allele frequencies less than 0.01 in 1,000 Genomes (October 2013), Genome Aggregation Database (gnomAD, https://gnomad.broadinstitute.org), the Exome Aggregation Consortium (ExAC; http://exac.broadinstitute.org), and the in-house database of DISCO study (>8,394 exomes) were extracted. Besides variants in *FBN2* gene, we also examined other variants according to American College of Medical Genetics and Genomics (ACMG) guidelines (Richards et al., 2015), The prediction of mutational effect was performed using Combined Annotation Dependent Depletion (CADD, https://cadd.gs.washington.edu/) (Rentzsch et al., 2019), Sorting Intolerant From Tolerant (SIFT, http://sift.jcvi.org/) (Ng and Henikoff, 2003), and PolyPhen-2 (http://genetics.bwh.harvard.edu/pph2/) (Adzhubei et al., 2013).

### CCA Clinical Score Calculation

We calculated the CCA clinical score of each affected individual based on their recorded phenotypes (Meerschaut et al., 2019). This scoring system was proposed to classify patients into likely CCA or unlikely CCA using phenotype-based scores. The scores were calculated based on the presence or absence of ten phenotypes. Four phenotypes were allotted for three points, including crumpled ears, arachnodactyly, camptodactyly and contractures of large joints. Two phenotypes were allotted for two points, including pectus deformity and dolichostenomelia. Four phenotypes were allotted for one point, including kyphoscoliosis, muscle hypoplasia, highly arched palate and micrognathia. A total score ≥7 and <7 indicated this patient was likely CCA or unlikely CCA, respectively. Only those with all the required phenotypes recorded were subjected for calculation.

## Results

### Clinical Presentation of the Ten Families

**FIGURE 1:**
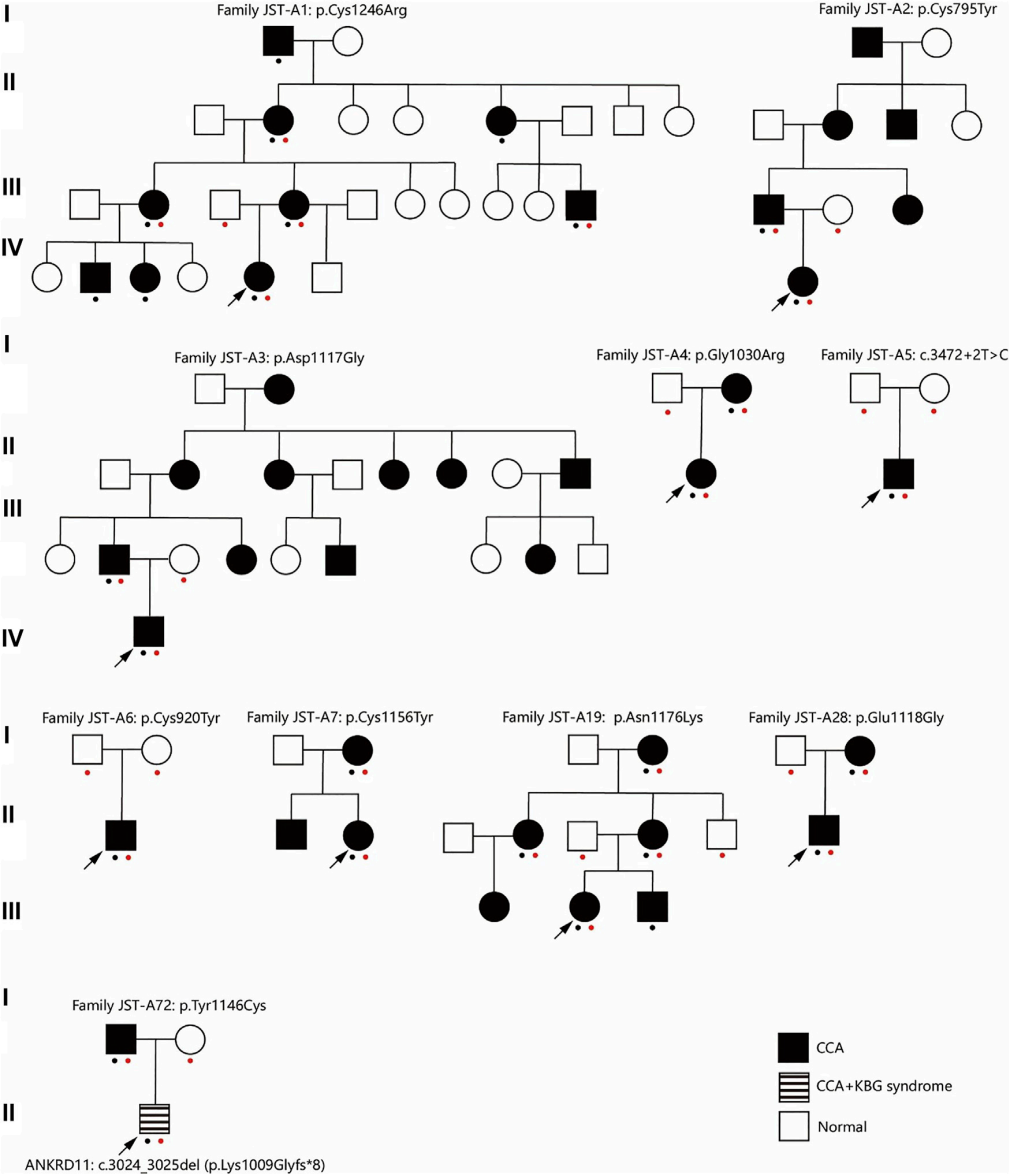
Pedigree of ten families. A red dot indicates this individual underwent genetic test (Sanger sequencing or exome sequencing). A black dot indicates this individual underwent phenotypes assessment. CCA, Congenital contractural arachnodactyly.

This study included 10 unrelated families with a total of 27 cases affected with CCA (Figure 1). Eight of the families have more than one affected individual. Median age at admission was 4.95 years. Arachnodactyly (27/27, 100%), crumpled ears (26/27, 96.3%), camptodactyly (26/27, 96.3%), and muscle hypoplasia (22/26, 85%) were observed in almost all recruited cases (Table 1). More than half patients (16/25, 64%) presented with contracture of large joints, including elbow, wrist, knee, ankle, and shoulder. 54% patients (13/24) present with kyphosis or scoliosis; 29% patients (7/24) presented with pectus deformity, including four patients with pectus carinatum and three patients with pectus excavatum; 74% patients (17/23) presented with high arched palate and 71% patients (17/24) presented with micrognathia; 33% patients (9/27) presented with pes planus (Table1); Two patients presented with genu varus and no patient presented with dolichostenomelia. Besides characteristic features of CCA, one patient presented with cryptorchidism, global developmental delay, atrial septal defect, bulbous nose, and broad eyebrow.

**TABLE 1:** Summary of patients’ phenotypes in our cohort and in the literature.

Phenotype	Our cohort	Literature
Crumpled ears	26/27 (96.3%)	37/45 (82%)
Arachnodactyly	27/27 (100%)	44/48 (92%)
Camptodactyly	26/27 (96.3%)	46/49 (94%)
Pectus deformity	7/24 (29%)	15/30 (50%)
Dolichostenomelia	0/20 (0%)	7/21 (33%)
Muscle hypoplasia	22/26 (85%)	17/24 (71%)
Large joints contracture	16/25 (64%)	37/45 (82%)
Micrognathia	17/24 (71%)	8/17 (41%)
Highly arched palate	17/23 (74%)	17/31 (55%)
Kyphoscoliosis	13/24 (54%)	31/43 (72%)

### Identification of *FBN2* Variants

After ES and bioinformatic analyses, ten different *FBN2* variants were identified from the ten families (Table 2). Two variants were validated to be *de novo* (Family JST-A5 and JST-A6) and eight variants were segregated with the phenotype. Of the ten variants, three have been previously reported, i.e. p.Tyr1146Cys, c.3472+2T>C and p.Asn1176Lys (Gupta et al., 2002; Callewaert et al., 2009). The remaining seven variants are all novel, i.e. p. Cys1246Arg, p. Glu1118Gly, p. Cys795Tyr, p. Asp1117Gly, p. Gly1030Arg, p. Cys920Tyr and p. Cys1156Tyr. All ten variants are evolutionary conserved and are absent from population controls.

**TABLE 2:** Summary of identified *FBN2* and *ANKRD11* variants.

Family ID	Gene	Nucleotide change	Protein change	Origin	ACMG Classification
Family JST-A1	*FBN2*	c.3736T > C	p.Cys1246Arg	M	LP
Family JST-A2	*FBN2*	c.2384G > A	p.Cys795Tyr	Pa	LP
Family JST-A3	*FBN2*	c.3350A > G	p.Asp1117Gly	Pa	LP
Family JST-A4	*FBN2*	c.3088G > A	p.Gly1030Arg	M	LP
Family JST-A5	*FBN2*	c.3472+2T > C	p.?	*de novo*	P
Family JST-A6	*FBN2*	c.2759G > A	p.Cys920Tyr	*de novo*	P
Family JST-A7	*FBN2*	c.3467G > A	p.Cys1156Tyr	M	LP
Family JST-A19	*FBN2*	c.3528C > A	p.Asn1176Lys	M	LP
Family JST-A28	*FBN2*	c.3353A > G	p.Glu1118Gly	M	LP
Family JST-A72	*FBN2*	c.3437A > G	p.Tyr1146Cys	Pa	LP
	*ANKRD11*	c.3024_3025del	p.Lys1009Glyfs*8	*de novo*	P

M, maternal; Pa, paternal; P, pathogenic; LP, likely pathogenic.

**FIGURE 2:**
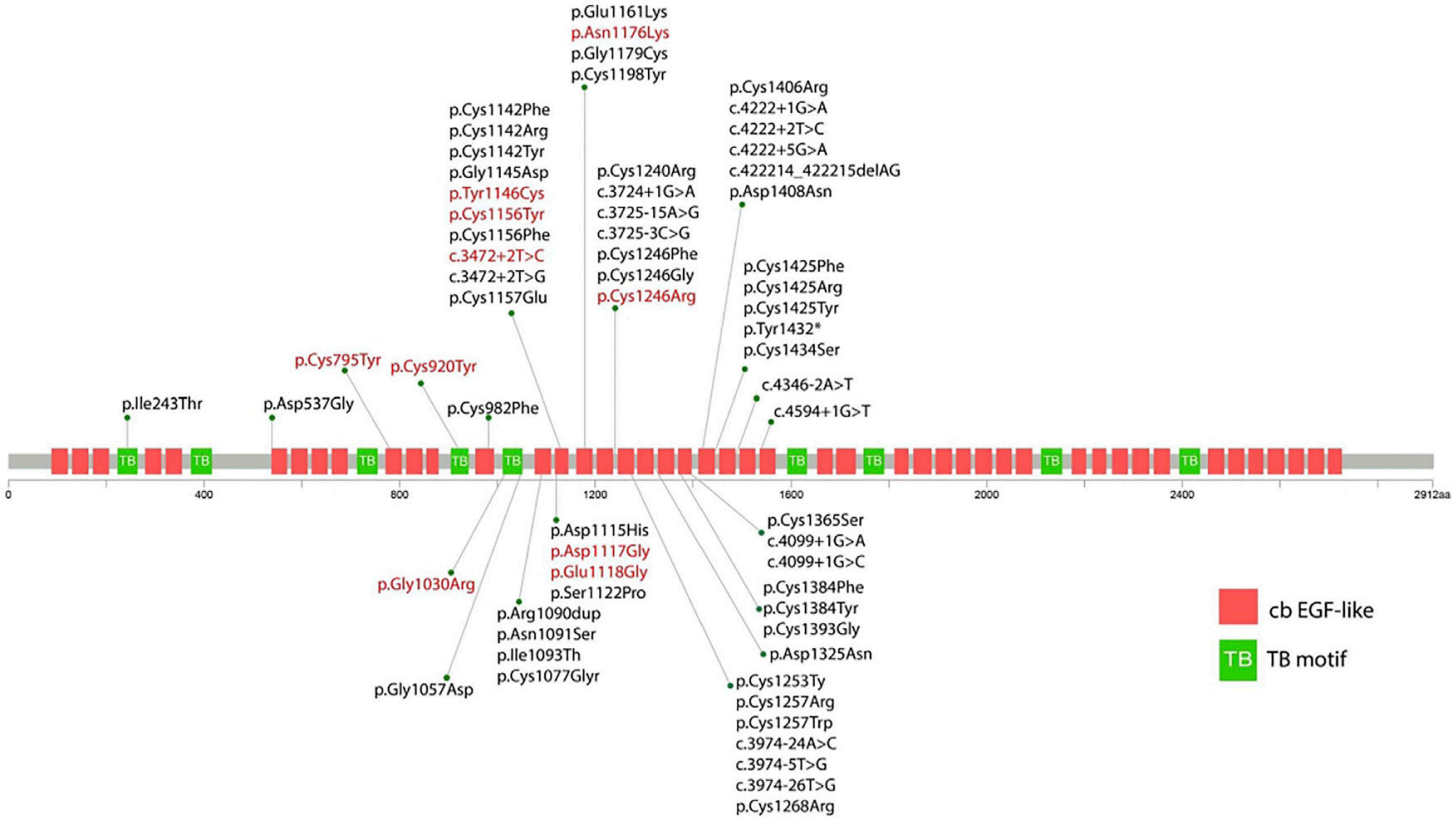
Schematic diagram showing the position of the identified variants in relation to the structural domains of the fibrillin-2 protein. The red variant indicates the variant reported in this study. The black variant indicates the variant reported in previous literature.

Eight out of ten variants were found in the neonatal region (exon 24-32) of the *FBN2* gene. Among them, six variants alter the highly conserved cbEGF-like domain (p.Tyr1146Cys, p. Asp1117Gly, p. Glu1118Gly, p. Cys1156Tyr, p. Asn1176Lys, and p. Cys1246Arg) (Figure 2). One variant alters the TB domain (p.Gly1030Arg). One *de novo* splicing variant was identified (c.3472+2T > C), which has been verified to leading to an exon 26 skip (Gupta et al., 2002). Two variants were found outside the neonatal region of the *FBN2* gene (p.Cys795Tyr and p. Cys920Tyr). The p. Cys920Tyr variant would affect the second hybrid domain and the p. Cys795Tyr would affect the cbEGF7 domain, respectively.

### Dual Diagnosis

Through interpretation of the ES data, we identified one patient with dual molecular diagnosis. Patient DISCO-JST A72 was a 5 months old boy born to nonconsanguineous northern Han parents. His father presented with typical features of CCA (crumpled ears, camptodactyly, arachnodactyly and dolichostenomelia), while his mother is healthy. At 5 months old, he presented with crumpled ears, camptodactyly, arachnodactyly, global developmental delay, short stature, cryptorchidism, intellectual disability, global developmental delay, atrial septal defect (3.5 mm), bulbous nose, and broad eyebrow. ES of this family reviewed a *de novo* heterozygous *ANKRD11* variant c.3024_3025del (p.Lys1009Glyfs*8) in addition to the heterozygous *FBN2* variant c.3437A > G (p.Tyr1146Cys) inherited from his affected father. The *ANKRD11* frameshift variant is previously unreported and predicted to result in protein truncation or nonsense medicated decay. Loss-of-function of *ANKRD11* gene is a known disease-causing mechanism (Kim et al., 2020). *ANKRD11* is the only known causative gene of KBG syndrome, which is characterized by macrodontia, craniofacial findings, short stature, multiple skeletal anomalies including vertebrae and limbs, neurologic involvement including global developmental delay, seizures, and intellectual disability (Goldenberg et al., 2016). The clinical diagnostic criteria of KBG syndrome included four features macrodontia of upper central permanent incisors, characteristic facial anomalies, hand anomalies, neurologic involvement, significantly delayed bone age, costo-vertebral anomalies, postnatal short stature, and a first degree relative with KBG. For this patient, we observed a remarkable blended phenotype. For global developmental delay, cryptorchidism, intellectual disability, atrial septal defect (3.5 mm), bulbous nose, and broad eyebrow, we considered these phenotypes to be part of KBG syndrome; while crumpled ears, camptodactyly and arachnodactyly are characteristic features of CCA.

### Validation of the CCA Scoring System

**FIGURE 3:**
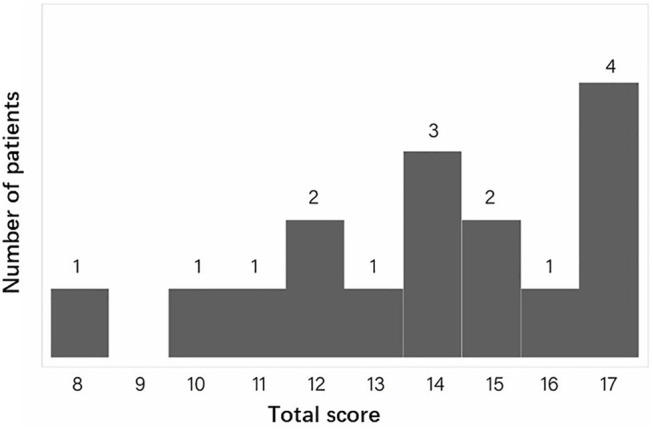
The distribution of the total CCA clinical scores for all eligible patients. The X ray indicates the total score. The Y ray indicates the number of patients.

We calculated the diagnostic score for CCA in our *FBN2*-positive CCA patients. Totally, 16 patients with sufficient phenotype data were eligible for analysis. Figure 3 gives an overview of the distribution of the total scores calculated for each CCA patient. The minimal, median and maximum score is 8, 14 and 17. All patients’ CCA score is ≥ 7, which indicates all patients are successfully classified into likely CCA by the CCA scoring system. For the rest of 11 patients with insufficient clinical data, the clinical score of these patients are still ≥7.

## Discussion

In this study, we investigated 10 families with 27 patients diagnosed with CCA, based on their clinical and molecular profiles. We identified seven novel variants and three previously reported variants in *FBN2* gene. Additionally, we reported an individual with dual molecular diagnosis of CCA and KBG syndrome.

*FBN2* encodes a 2912-amino acids extracellular matrix protein related to the elasticity of the tissue, which includes nine TB motifs, and 46 cbEGF domains (Davis and Summers, 2012). Each EGF-like domain contains six conserved cysteine residues to support its native folding. Six conserved cysteine residues form three disulfide bridges to maintain protein stability (Davis and Summers, 2012). In the present study, we found that c.2384G > A (p.Cys795Tyr), c.2759G > A (p.Cys920Tyr), c.3437A > G (p.Tyr1146Cys), c.3467G > A (p.Cys1156Tyr), and c.3736T > C (p.Cys1246Arg) alter or produce cysteine residues in the cbEGF domain, which would potentially disrupt the disulfide bond and therefore impair the nature folding of fibrillin-2. This is considered as the major mechanism underlying the pathogenesis of CCA (Guo et al., 2016; Xu et al., 2020).

Nine out of ten variants reported in this study are missense variants, which is consistent with previous findings that nearly all currently reported human *FBN2* pathogenic variants lead to single amino acids substitutions or in-frame exon deletions or duplications. This observation strongly suggests that gain-of-function is the key mechanism underlying the pathogenesis of CCA. This might explain why *fbn2* null mice does not phenocopy human CCA (Shi et al., 2013; Sengle et al., 2015).

In our cohort, we detected only two pathogenic variant [c.2384G > A (p.Cys795Tyr) and c.2759G > A (p.Cys920Tyr)] located outside the neonatal region. There are only three variants outside the neonatal region have been previously reported to lead to CCA (Liu et al., 2019; Renner et al., 2019). Totally, only ∼8% CCA was caused by variants outside the neonatal region, which proves from another side of the substantial contribution of the neonatal region to the CCA phenotype. The pathogenicity of these two variants were supported by *in silico* predictions of pathogenicity, the absence of this variant from population controls, and segregation analysis.

We found one case with a blended phenotype consisting of CCA and KBG syndrome. Dual diagnoses in Mendelian disorders with complex phenotypes are being increasingly recognized. Coexisting diseases result in blended clinical phenotypes and poses challenge in diagnosis and management (Posey et al., 2017). One study have found 4.9% of cases suspected of having Mendelian disorders had multiple molecular diagnoses (Posey et al., 2017). Therefore, when phenotypes cannot be completely explained by one detected variant, additional genetic and clinical assessment should be considered. Interestingly, patients with CCA typically presented with tall stature, while KBG syndrome usually resulted in postnatal short stature. In this case, the patient has remarkable short stature.

Review of the phenotype data of CCA probands from literature and our cohort reveals a basically comparable phenotype. For example, external ear malformations like crumpled ears, which are a major characteristic of CCA, were found in 96.3% and 82% patients in our cohort or in the literature, respectively. The high prevalence of crumpled ears in CCA patients suggest it is particularly important in the differential diagnosis of connective tissue disorder. Interestingly, we found no patients in our cohort presented with dolichostenomelia. While in previous reports, 7/21 (33%) patients presented with dolichostenomelia (Fisher’s exact test, *p* = 0.0005). Additionally, we found no patients in our cohort presented with cardiovascular anomalies like aortic root dilation. Our findings suggest phenotype heterogeneity of CCA may exists in different populations.

Genotype-phenotype correlation analysis in this study and previous studies revealed no significant associations. Recently, mutational burden has been recognized has a key contributor to the molecular diagnosis of some patients with arthrogryposis through accumulation of multiple deleterious variants (Pehlivan et al., 2019). Multilocus variants might also contribute to the genotype-phenotype correlation and intrafamilial variability in CCA. Fibrillins-2 polymerize extracellularly and form microfibrils with many proteins, e.g., latent transforming growth factor beta binding proteins (LTBPs) and microfibril-associated proteins (MFAPs). The complex binding interactions between these molecules indicate variants in different genes could modify CCA phenotype. Further analysis of large samples would possibly provide insights into the genotype-phenotype correlation in CCA.

The newly developed clinical scoring system for CCA successfully classified all patients in this study into likely CCA, even in patients with insufficient clinical data, indicating the excellent sensitivity of this scoring system. However, we didn’*t* test the specificity of this scoring system due to lack of comprehensive clinical data of possibly misdiagnosed syndromes like Marfan syndrome. Marfan syndrome usually presented with cardiovascular, skeletal and ophthalmological manifestations (Bitterman and Sponseller, 2017). The overlapping phenotypes mainly include scoliosis, pectus deformity and cardiovascular deformity. In this scoring system, these overlapping phenotypes like kyphoscoliosis and pectus deformity were allotted for three points in total. While large joints contractures, camptodactyly, ear malformation, and arachnodactyly are usually absent in Marfan syndrome, which were allotted for three points each. Thus, we can speculate this scoring system would likely diagnose a Marfan syndrome patient into “unlikely CCA”. Nevertheless, more data are needed to validate the specificity of this scoring system.

Intrafamilial heterogeneity has been noted in some families with CCA. This phenomenon has also been observed in our cohort and showed by the CCA clinical score. In the family JST-A1 with eight patients eligible for analysis, the highest, lowest and median CCA clinical score was 17, 8 and 13, respectively. The intrafamilial variation in this large family was moderate and all patients were classified into “likely CCA”.

In conclusion, we report seven novel and three previously reported *FBN2* mutations in 27 patients from ten families with CCA. Our report enriches the mutational spectrum of *FBN2* and validate the novel CCA scoring system.

## Data Availability

The datasets presented in this study can be found in online repositories. The names of the repository/repositories and accession number(s) can be found below: <https://data.mendeley.com/drafts/cxgcj3s8t3, Mendeley.
