# Supplementary material for: Identification of Novel FBN2 Variants in a Cohort of Congenital Contractural Arachnodactyly
Source: Front Genet. 2022 Mar 10;13:804202. doi: 10.3389/fgene.2022.804202 (PMC8960307; doi:10.3389/fgene.2022.804202)
Supplement: Supplementary file 1 [file Table1.pdf]

Table S1. Detailed phenotypes of each patient.

1, present; 0, absent; NA, not available.

Pedigree	Crumpled ears	Arachnodactyly	Camptodactyly	Contractures of large joints	Pectus deformity	Dolichostenomelia	Kyphoscoliosis	Muscle hypoplasia	Highly arched palate	Micrognathia
**Family JST-A1**	1	1	1	1	1	0	1	1	1	1
	1	1	1	1	1	0	1	1	1	1
	1	1	1	1	0	0	1	1	1	0
	1	1	1	1	0	0	0	1	0	0
	1	1	1	1	0	0	0	1	0	0
	1	1	1	1	0	0	1	1	1	0
	1	1	1	1	0	0	1	1	1	0
	1	1	1	1	1	0	1	1	1	1
**Family JST-A2**	1	1	1	1	1	0	0	1	0	1
	1	1	1	0	0	0	0	1	0	1
**Family JST-A3**	1	1	1	1	0	0	1	1	1	1
	1	1	0	1	0	0	0	0	1	1
**Family JST-A4**	1	1	1	1	0	0	0	1	1	1
	1	1	1	0	0	0	0	0	1	1
**Family JST-A5**	1	1	1	NA	0	NA	0	NA	NA	NA
**Family**	1	1	1	0	0	NA	1	0	0	1

**JST-A6**										
**Family JST-A7**	1	1	1	1	NA	0	NA	1	1	1
	1	1	1	0	NA	0	NA	1	1	1
**Family JST-A19**	1	1	1	1	1	0	1	1	0	1
	1	1	1	1	1	0	1	1	1	1
	1	1	1	0	0	NA	1	1	1	0
	1	1	1	0	0	NA	1	1	1	0
	1	1	1	0	0	NA	NA	1	NA	1
**Family JST-A28**	1	1	1	1	NA	0	0	1	1	1
	1	1	1	0	1	0	1	1	1	1
**Family JST-A72**	1	1	1	0	0	NA	0	1	NA	NA
	0	1	1	NA	0	NA	0	0	NA	NA
